# SH005S7 Overcomes Primary and Acquired Resistance of Non-Small Cell Lung Cancer by Combined MET/EGFR/HER3 Inhibition

**DOI:** 10.1155/2022/1840541

**Published:** 2022-09-15

**Authors:** Sooyeon Kang, Ji Hye Kim, Jisoo Hong, Ji Hong Moon, Yun Young Kwon, Seong-Gyu Ko

**Affiliations:** ^1^Department of Science in Korean Medicine, Graduate School, Kyung Hee University, Seoul 02447, Republic of Korea; ^2^Department of Preventive Medicine, College of Korean Medicine, Kyung Hee University, Seoul 02447, Republic of Korea

## Abstract

In this study, we have examined the anticancer effects of SH005S7 on MET-amplified and (HCC827GR) NSCLC cells and their primary HCC827 cells. *In vitro*, first of all, cell viability and colony formation assay confirmed the growth inhibitory effects of SH005S7 on both cells. Second, SH005S7 inactivated EGFR-related multiple cell signaling, which was associated with a marked decrease in the constitutive phosphorylation of EGFR, HER3, MET, AKT, and ERK. Third, SH005S7 attenuated the anchorage-independent cell growth. Fourth, SH005S7 blocked invasive and metastatic capability by downregulation of mesenchymal markers—vimentin, snail, and MMP-9. Fifth, BrdU assay confirmed the cell cycle arrest of SH005S7 on these cells. When administered orally to nude mice xenografically transplanted human NSCLC, SH005S7 inhibited the growth of tumor and did not cause hepatotoxicity and nephrotoxicity in animals. Immunohistochemical and Western blot analyses of tissue showed that the suppression of growth correlated with inhibition of proliferation (Ki-67, PCNA), invasiveness (vimentin, snail), and angiogenesis (CD31) marker and decrement in the constitutive and phosphorylation of EGFR, HER3, MET, AKT, and ERK. Additionally, SH005S7 had immune stimulatory effects by TNF-*α* cytokine release on macrophage, without cell cytotoxicity. Overall, our results suggest that SH005S7 can inhibit the growth of MET-amplified and gefitinib-resistant NSCLC cells through the suppression of EGFR-related multiple targets linked to overcome gefitinib resistance.

## 1. Introduction

Despite surgical and/or chemotherapeutic treatments, lung cancer is still the leading cause of cancer-related death in the WHO [1]. Non-small cell lung cancers (NSCLC), about 4/5 cases of all lung cancers, have multiple mutations in the tyrosine kinase domain of epidermal growth factor receptor (EGFR) gene; therefore, many tyrosine kinase inhibitors (TKIs) have been developed as chemo- and target therapeutic agents for the treatment of NSCLC [2–6]. However, these successful therapeutic regimens have been reported that the acquired chemoresistance against TKIs treated patients within a few years [7, 8]. Acquired drug-resistant patients have been shown to be correlated with a secondary T790M gatekeeper mutation in EGFR and activation of mesenchymal epithelial transition factor (MET) signaling by MET amplification [9–12].

The EGFR family is widely expressed in the cell surface as receptors, and mutation of it has been shown to be associated with poorer clinical results in chemotherapeutic resistance in NSCLC. This family consists of four distinct, but structurally similar, homologous members: c-ERBB1/EGFR/EGFR1 (commonly referred to as EGFR), c-ERBB2/HER2 (commonly referred to as HER2), c-ERBB3/HER3, and c-ERBB4/HER4 [13–15]. The individual RTK family signaling molecules initiate several signal transduction cascades, mainly the mitogen-activated protein kinase/extracellular signal-regulated kinase (MAPK/ERK) and phosphoinositide-3-kinase/protein kinase B (PI3K/AKT) pathways, leading to DNA synthesis, survival, cell proliferation, angiogenesis, tumor cell motility, invasion, and metastasis in the cancer [16–22].

The MET, which originally was identified a protooncogene, was later found to be a transmembrane RTK activated by a ligand such as the hepatocyte growth factor (HGF) [23–26]. Dysregulation of MET signaling-mediated proliferation, cell death, and migration through overexpression of MET protein and amplification or mutation of the MET gene has been demonstrated in oncogenic processes across several tumor types and has been steadily reported [27–31]. Moreover, it is particularly notable that mechanisms of MET/MET signaling dysregulation have been reviewed in NSCLC [32–35]. Both EGFR and MET are widely expressed on tumor cells, and these RTKs are implicated in diverse tumor progression signaling processes. Therefore, a rational strategic therapy, combining various standard therapies with MET and/or EGFR inhibitors, has been explored in the preclinical or clinical studies to develop drugs overcoming acquired chemotherapy resistance [36, 37].

Natural products have been used as medicine from the ancient times, and 70% of all drugs approved for cancer treatment between 1981 and 2002 were either natural products or based on natural products [38–41]. SH005S7, a new herbal formula, was composed of *Rhus verniciflua* Stokes (RVS), *Angelica gigas* Nakai (AGN), and *Morus alba* L. (MAL). This remedy is modified from “jeungmisamultang” introduced by the Dongui Bogam, which is the excellent Korean medicine book written by the royal physician. RVS has been used for the treatment of gastric problems, hepatic disorders, infectious diseases, and blood disorders in Eastern Asia. Modern science has provided the scientific basis for the use of RVS against inflammatory diseases, neurodegenerative diseases, and various cancer types in *in vitro*, *in vivo*, and human studies [42]. AGN is also used as a traditional medicinal herb in East Asian countries. It has been reported to have anticancer, neuroprotective, prevention of obesity, and anti-inflammatory effects *in vitro* and *in vivo* [43–45]. MAL, the root bark of *Morus alba* L., has been traditionally used for antiphlogistic, diuretic, antitussive, and cough treatment in oriental medicine [46]. Modern science has provided that MAL has immunological, antihypolipidemic, antioxidant, anti-inflammatory, antibacterial, and antiviral activities in cell lines and animal study [47–52]. Many researches have been conducted to treat lung cancers with each traditional medicine; however, no study has been carried out to examine anticancer potential of a new remedy until now.

The primary objective of this study was to determine whether SH005S7 has anticancer potential by overcoming primary and acquired resistance of lung cancer cells *in vitro* and *in vivo*. If so, we sought to determine the mechanism by which SH005S7 modulates these effects.

## 2. Materials and Methods

### 2.1. Preparation of SH005S7

The SH005S7 herbal formula was composed of RVS, AGN, and MAL at a 2 : 1 : 1 ratio, which is a modified prescription of traditional Korean medicine. The root bark of MAL and the root of AGN were supplied from Jaseng Hospital of Korean Medicine (Seoul, Republic of Korea) and manufactured following the guidelines of the herbal Good Manufacturing Practice (hGMP). The root bark of RVS was provided by Kyung Hee University Hospital (Seoul, Republic of Korea). RVS and AGN were prepared by distilled water (D.W.) and MAL was extracted by 30% ethanol. HPLC analysis was performed for qualification of herbal mixture including each component and was carried out in a Hanpoong Pharm and Foods Company (Jeonju, Republic of Korea) manufactured by the Korea Good Manufacturing Practice (KGMP) and a Korean Medicine Clinical Trial Center of Kyung Hee University Korean Medicine Hospital (Seoul, Republic of Korea).

### 2.2. HPLC

Chromatographic analysis of SH005S7 and standard compound fisetin were determined by HPLC and 5 *μ*m column (Capcell Pak® C18, 250 × 4.6 mm, Phenomenex, Torrance, USA). Using mobile phases A (acetonitrile : methanol : tetrahydrofuran : water in a ratio of 19 : 5 : 1 : 75, pH 3) and B (acetonitrile : methanol : water in a ratio of 55 : 15 : 30), the gradient program was run as follows: 15 min (2% B), 28 min (28% B), and 40 min (36% B) at 1 mL/min flow velocity and 360 nm wavelength. Chromatographic analysis of decursin content in SH005S7 was performed by HPLC column (inner diameter 4–6 mm and length 15–20 cm, 5 *μ*m, octadecylsilylated silica gel in a stainless tube), and the separation was carried out using mobile phase acetonitrile : water (13 : 12). The injected sample was then analyzed for decursin at 0.7 mL/min flow velocity and 280 nm wavelength. The SH005S7 extract and standard compound morusin were quantitatively analyzed as follows. The dried extract, with approximately 0.1 g SH005S7, was dissolved in 70 mL of ethanol (JT Baker, HPLC grade, Philipsburg, NJ, USA). The standard material was weighed, 2 mg, and dissolved in methanol, 2 mL (JT Baker, HPLC grade, Philipsburg, NJ, USA). The test sample was analyzed by HPLC with a XSelect HSS T3 column (150 mm × 4.6 mm, 3.5 *μ*m column, Waters, Milford, USA). Using mobile phases A (water) and B (Methanol), the gradient program was run as follows: 15 min (2% B), 28 min (28% B), and 40 min (36% B) at 1 mL/min flow velocity and 270 nm wavelength. The injected sample was then analyzed for morusin at 0.7 mL/min flow velocity and 280 nm wavelength. Amounts of standard compounds (fisetin, decursin, and morusin) were calculated from area under curve (AUC).

### 2.3. Cell Lines and Cell Culture Conditions

Human lung adenocarcinoma, primary HCC827, and gefitinib-resistant HCC827GR cells were kindly provided by Dr. Sung Keun Jung, division of functional food research, Korea Food Research Institute, Jeollabuk-do. The mouse macrophage cell line RAW 264.7 was purchased from the Korean Cell Line Bank (KCLB) (Seoul). Human lung cancer cells were cultured in RPMI 1640 (Welgene, Gyeongsan, Republic of Korea) with 10% heat-inactivated FBS (J R Scientific, Woodland, CA, USA) and 100 units/mL penicillin-streptomycin (Lonza, Basel, Switzerland). RAW264.7 cells were grown in DMEM supplemented with 10% FBS and 1% penicillin-streptomycin. Cells were incubated in a humidified atmosphere with 5% CO_2_ at 37°C.

### 2.4. Western Blot

Whole-cell extracts of untreated and treated cells were used in Western blot analysis as described previously [53]. Antibodies against p-EGFR (cat no. 3777; 1 : 1000), EGFR (cat no. 2232; 1 : 1000), p-MET (cat no. 3077; 1 : 1000), MET (cat no. 8198; 1 : 1000), p-HER3 (cat no. 4791; 1 : 1000), HER3 (cat no. 12708; 1 : 1000), p-AKT (cat no. 9271; 1 : 1000), AKT (cat no. 9272; 1 : 1000), GAPDH (cat no. 5174; 1 : 1000), vimentin (cat no. 5741; 1 : 1000), snail (cat no. 3879; 1 : 1000), and MMP-9 (cat no. 13667; 1 : 1000) were from Cell Signaling Technology (Danvers, Massachusetts, USA). P-ERK1/2 (cat no. sc7383; 1 : 1000) and ERK2 (cat no. sc1647; 1 : 1000) antibodies were purchased from Santa Cruz Biotechnology (Dallas, Texas, USA).

### 2.5. Cell Viability Assay

The effect of SH005S7 on cell proliferation was determined by the 3-(4,5- dimethylthiazol-2-yl)-2,5-diphenyltetrazolium bromide (MTT) uptake method as described previously [54].

### 2.6. Colony Formation Assay and Anchorage-Independent Growth Assay

For colony formation assay, cells (5 × 10^2^/well) were seeded in 6-well plates and treated to indicated concentrations of SH005S7 (0, 50, 100 *μ*g/mL) for 14 days. When colonies began to appear, 0.25% crystal violet solution was dispensed in a well of 1 mL and stained for 30 minutes. For anchorage-independent growth assay, after 0.5% bottom layer of agar was completely hardened, lung cancer cells were seeded in a 6-well plate at a density of 5 × 10^3^ cells per well in 0.3% top layer of agar mixed with SH005S7. The fresh medium was added to wells twice a week. Colonies were captured after about 3 weeks under a microscope (Olympus, Tokyo, Japan).

### 2.7. Cell Invasion and Wound Healing Assay

For invasion assay, on day 1, cells were grown in serum-free medium for 18–24 hours. On day 2, the upper chamber with suspension of Matrigel (Corning, Corning, NY, USA) and coating buffer (0.01 M Tris (pH 8.0) and 0.7% NaCl, filtered using a 0.2 *μ*m sterile filter unit) was incubated for 2 hours in a 5% CO_2_ at 37°C condition. After removing the remaining gel in transwell insert of upper compartment, cells (2 × 10^5^ cells per chamber) were plated in transwell insert with SH005S7 and chemoattractant was added to the lower compartment. On day 3, the membrane was swabbed to remove the noninvasive cells in the upper chamber and the membrane was stained with 1% crystal violet. The solution obtained by dissolving stained cells in 1% acetic acid was measured at 560 nm using an ELISA reader. For wound healing assay, cells (5 × 10^5^ cells/well) were cultured in a 6-well plate to obtain 90% confluency. A wound was drawn with a 200 *μ*L pipette tip and immediately treated with SH005S7. Micrographs were captured at 0 and 48 hours.

### 2.8. BrdU Assay

Cells were treated with SH005S7 for 72 hours and then incubated in the 10 *μ*M BrdU labeling solution for 1 hour at 37°C in a CO_2_ incubator. Cells were harvested by centrifugation at 400 g for 5 minutes and fixed in 100% ethanol on ice for 20 minutes before storage at −20°C or direct analysis. Next, cells were incubated in 2N HCl/0.5% Triton X-100 for 30 minutes at room temperature (RT). Acid was neutralized by dispensing 0.1 M sodium tetraborate (pH 8.5) into the cell suspension. After washing with PBS/1% BSA, cells were incubated at RT for 30 minutes by adding anti-BrdU antibody (cat no. sc32323, Santa Cruz) to the solution which is made of 5% Tween 20 in 1% BSA/PBS. Cells were washed and then incubated for 30 minutes at RT with 1 : 100 goat anti-mouse IgG-FITC (Santa Cruz). Cells were resuspended in 5 *μ*g/mL PI for 30 minutes on ice. FACS analysis was then performed (342973, BD FACSCalibur™, San Jose, CA, USA).

### 2.9. Generation of Bone Marrow-Derived Macrophages (BMDMs)

BMDMs were generated from the bone marrow (BM) cells of 7-week-old male BALB/c mice. All animals underwent 7 days of adjustment prior to experiments. BM cells were harvested from femurs and tibiae of mice, which flushed with R10 based on the RPMI-1640 medium containing 10% FBS, 1% penicillin-streptomycin, and 0.05 mM 2-mercaptoethanol. Red blood cells were removed by adding red blood cell (RBC) lysis buffer (cat no. 11814389001, Roche, Basel, Switzerland), and the remaining cell pellet was diluted in R10. The number of viable cells was counted using a trypan blue solution (cat no. 93595, Merck Millipore, Burlington, MA, USA). The cell suspension was cultured at a concentration of 2 × 10^6^ cells/100*π* dish in complete R10 adding 20 ng/mL recombinant murine granulocyte-macrophage colony-stimulating factor (GM-CSF) (cat no. 315-03, PeproTech, Rocky Hill, NJ, USA). At day 3, another fresh R10 containing 20 ng/mL GM-CSF was added. At days 6 and 8, half of the culture supernatant was collected and centrifuged and the cell pellet was resuspended in fresh R10 containing 20 ng/mL GM-CSF and given back into the original plate. At day 10, adherent BMDMs were harvested by a cell scraper and pooled for subsequent in experiments.

### 2.10. Mouse Cytokine Array

Cells were stimulated with SH005S7 at the indicated concentrations for 48 hours. The cell culture medium was collected and then stored at −80°C. The supernatants were tested for cytokine expression profiles, using the mouse cytokine array panel A kit (R&D Systems, Minneapolis, Minnesota, USA) according to the manufacturer's instructions.

### 2.11. Xenograft Mouse Model

Six-week-old male BALB/c (nu/nu) mice were obtained from NARA Biotech (Seoul, Republic of Korea) and maintained under specific pathogen-free conditions for the duration of the experiment. Mice were divided into two groups: (1) vehicle group (*n* = 5) and (2) 500 mg/kg of SH005S7 group (*n* = 6). HCC827GR cells (2 × 10^6^ cells/200 *μ*L) were suspended in PBS mixed with growth factor-reduced Matrigel (1 : 1) and inoculated subcutaneously into the right flank of each mouse. Tumor volume was calculated using the following formula: (*π*/6 × *L* × *W*^2^ and 1/2 × *L* × *W*^2^). Mice were incubated until the tumor volume reached 1 cm^3^ and were sacrificed at that time.

### 2.12. *In Vivo* CBC and Serum Analysis

Complete blood cells (CBC) were measured using a hematology analyzer (Drew Scientific, Miami-Dade County, FL, USA). The serum biochemical profile was obtained from the automated clinical chemistry analyzer (Fujifilm, Tokyo, Japan). Analyzers were performed as recommended by the manufacturer.

### 2.13. Immunohistochemistry (IHC)

Paraffin-embedded tissue sections (5 *μ*M) were deparaffinized in xylene (Sigma-Aldrich) and rehydrated with ethanol (Merck Millipore). The sections were subsequently incubated with PBS/5% BSA, followed by an overnight incubation at 4°C with the primary antibodies (100–200 *μ*L; rabbit monoclonal anti-p-EGFR, EGFR, p-MET, MET, p-HER3, HER3, vimentin (1 : 10), and Ki-67 (cat no. ab16667; 1 : 50, Abcam, Cambridge, UK); rabbit polyclonal anti-CD31 (cat no. ab28364; 1 : 10, Abcam); and mouse monoclonal anti-PCNA (cat no. upstate 05-347; 1 : 10, Merck Millipore)). The tissue sections were visualized using an ABC kit (Vector Laboratories, Burlingame, CA, USA) and developed using a diaminobenzidine (DAB Kit; Vector Laboratories). The mounted slides were observed under an Axio Observer (Carl Zeiss, Oberkochen, Germany). The staining intensities of the primary antibodies were done using ImageJ software. The values were subjected to two-tailed Student's *t*-test.

### 2.14. Hematoxylin and Eosin (H&E) Staining

Sections were stained with hematoxylin for 10 minutes followed by eosin for 1 minute and 30 seconds, hydrated back to H_2_O, and then covered with coverslips using a mounting medium (Thermo Fisher Scientific, Waltham, MA, USA).

### 2.15. Statistical Analysis

All data analysis was conducted through Microsoft Excel software and was performed with the two-tailed Student *t*-test. The *p* value was considered as significant differences (^∗^*p* < 0.05, ^∗∗^*p* < 0.01, and ^∗∗∗^*p* < 0.001).

## 3. Results and Discussion

### 3.1. Results

#### 3.1.1. SH005S7 Contains RVS, AGN, and MAL and Inhibits the RTK-EGFR, MET, and HER3 in Gefitinib-Resistant Lung Cancer Cells

**Figure 1:**
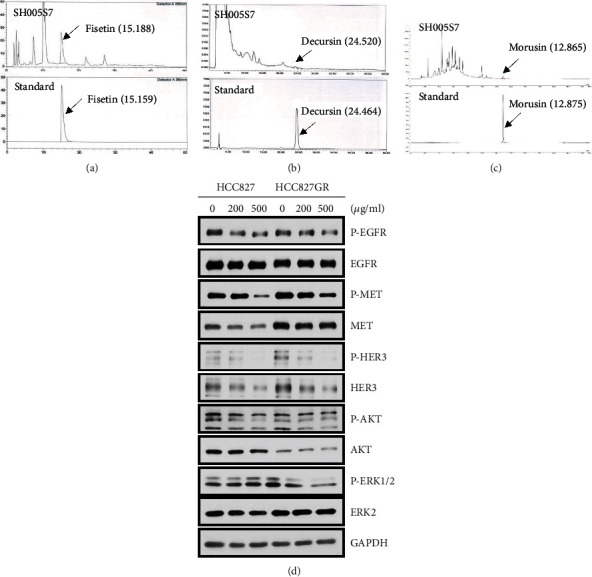
Determination of index compounds in SH005S7 by HPLC analysis and the effect of SH005S7 downregulating the RTK signaling pathway in gefitinib-resistant lung cancer cells. Peaks of standard compounds were identified as shown. (a) Characterization of fisetin in the SH005S7 formula (top panel) using pure fisetin as standard (lower panel). (b) Decursin in SH005S7 (top panel) using pure decursin as standard (lower panel). (c) Morusin in SH005S7 (top panel) using pure morusin as standard (lower panel). (d) SH005S7 suppresses expression of EGFR, MET, and HER3 in both HCC827 and HCC827GR cells. Cells were treated with vehicle, 200, and 500 *μ*g/mL SH005S7 for 72 hours. The results from three independent experiments.

The characterization of the SH005S7 herbal formula requires standard compounds. The SH005S7 formula consists of three different herbs, RVS, AGN, and MAL. We analyzed the amount of standard compound present in SH005S7 (Figures 1(a)–1(c)). The HPLC analysis indicated that SH005S7 contained 1.3% fisetin, 0.05% decursin, and 0.019% morusin (Table 1). As expected, gefitinib treatment significantly inhibited cell viability in primary HCC827 cells, but not in gefitinib-resistant HCC827GR cells (Supplementary Figure 1(a)). In HCC827 cells, gefitinib inhibited phosphorylation of EGFR, MET, and HER3 and subsequent activation of the cell survival signaling pathway in a dose-dependent manner but had no changes in HCC827GR cells (Supplementary Figure 1(b)). However, SH005S7 showed the same inhibitory effects on phosphorylation of RTKs (EGFR, MET, and HER3) in HCC827 as well as HCC827GR cells (Figure 1(d)).

#### 3.1.2. SH005S7 Suppresses Cell Growth of Lung Cancers

**Figure 2:**
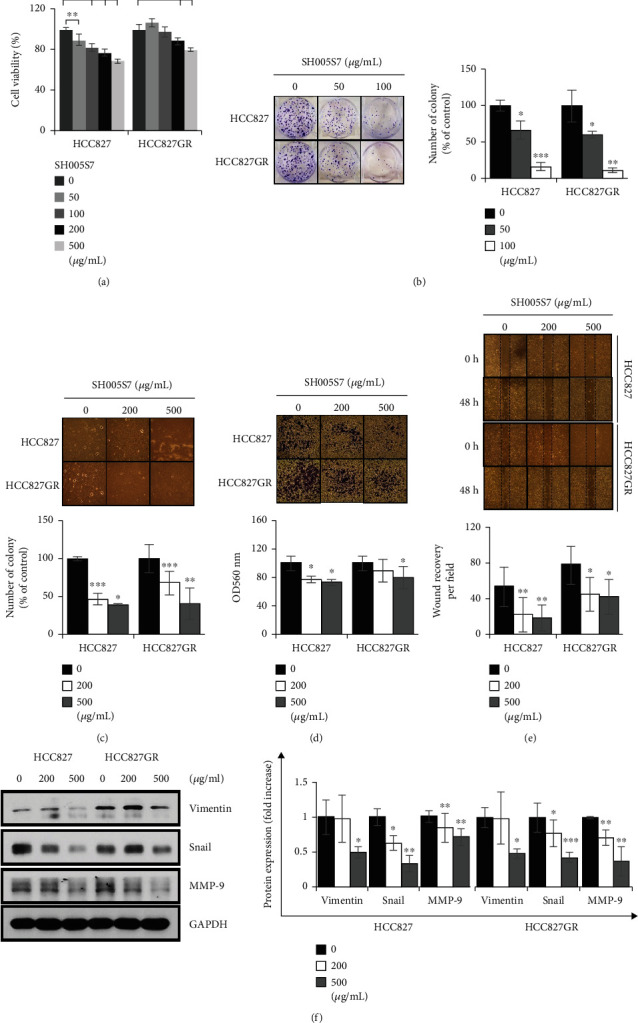
SH005S7 exhibits anticancer effects on lung cancer cells. (a) SH005S7 inhibits cell proliferation of HCC827 and HCC827GR cells. (b) SH005S7 restrained colony formation of both HCC827 and HCC827GR cells, and the effects were significant at lower concentrations of SH005S7 (50, 100 *μ*g/mL) for 7 days. (c) SH005S7 impeded anchorage-independent cell growth. Random areas were scanned (three areas per membrane of the well) in cells, and error bars represent standard deviation (S.D.) of three areas. SH005S7 prevents invasion and migration of lung cancer cells in the (d) Matrigel invasion assay or (e) wound healing assay. After incubation for 24 hours with the indicated concentrations of SH005S7, cells that invade to the lower surface were fixed, stained, and measured using an ELISA reader. Following to 48 hours with SH005S7 treatment, cells that migrate to the wound surface were estimated using microscopy. (f) SH005S7 inhibits the protein expression of EMT markers in HCC827 and HCC827GR cells. Data shown are the representative of three independent experiments (error bars are S.D. (^∗^*p* < 0.05, ^∗∗^*p* < 0.01, and ^∗∗∗^*p* < 0.001 versus control)).

Because SH005S7 suggested to be a potential RTK inhibitor in primary and gefitinib-resistant lung cancer cells, we hypothesized that SH005S7 may have a function to inhibit cell growth. We first assessed *in vitro* proliferation and colony formation assays. In the MTT assay, the HCC827 and HCC827GR cells showed reduced viability in a dose-dependent manner (Figure 2(a)). In the colony formation assay, the number of colonies from the SH005S7-treated cells was lesser than that from nontreated cells (Figure 2(b)). Then, we examined the effects of SH005S7 on the anchorage-independent growth of lung cancer cells using the soft agar assay. The number of anchorage-independent colonies of the SH005S7-treated cells was significantly reduced as compared with that of the control cells (Figure 2(c)). These results suggest that SH005S7 induced inhibition of cell proliferation regardless of gefitinib resistance. Growing cancer cells will eventually move to other tissues through invasion and metastasis, which is involved in cell growth, cell survival, and tumor cell development. In order to investigate the effects of SH005S7 on the invasiveness of lung cancer cells, we explored cell invasion and migration assays using HCC827 and HCC827GR cells. SH005S7 suppressed both invasion and metastasis capacities in a dose-dependent manner (Figures 2(d) and 2(e)). Also, the protein levels of mesenchymal markers vimentin, snail, and MMP-9 were downregulated in the SH005S7-treated lung cancer cells (Figure 2(f)). These results indicate that the invasive growth of HCC827 and HCC827GR cells was attenuated by SH005S7 treatment.

#### 3.1.3. SH005S7 Induces S Phase Cell Cycle Arrest

**Figure 3:**
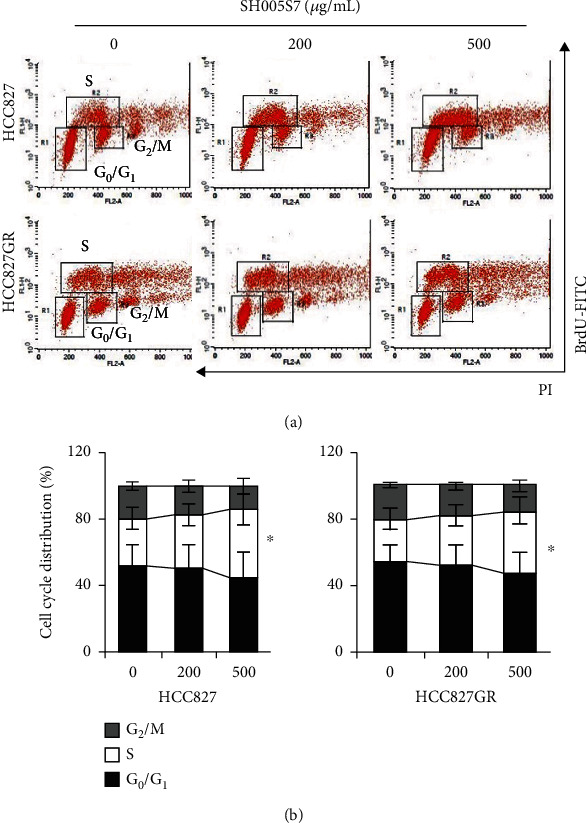
SH005S7 restrains cell cycle progression in HCC827 and HCC827GR cells. (a) FACS analysis of HCC827 (top panels) or HCC827GR (lower panels) cells treated with SH005S7 for 72 hours after labeling with BrdU for 1 hour, followed by staining with propidium iodide (PI). The BrdU incorporation (*y*-axis) was monitored by anti-BrdU antibody, and DNA contents were monitored by PI (*x*-axis). (b) The proportion of BrdU-positive cells was determined and expressed relative to untreated controls. ^∗^ Indicates that the values were significantly different (^∗^*p* < 0.05) by Student's *t*-test. The results from three independent experiments.

To determine the effects of SH005S7 on cell cycle distribution in HCC827 and HCC827GR, we performed two-parameter flow cytometry to the more clearly separate S phase from other cell cycles by analyzing BrdU incorporation and DNA contents. After exposure to SH005S7 for 72 hours, we observed a substantial decrease in BrdU-negative cells with 2N (G1 phase) and 4N DNA contents (G2/M phase). On the contrary, BrdU-positive cells gradually increased in both cell lines in a dose-dependent manner (Figure 3). This demonstrates that SH005S7 induces S phase arrest.

#### 3.1.4. SH005S7 Has Immune Stimulatory Effects by Cytokine Release on Macrophage

**Figure 4:**
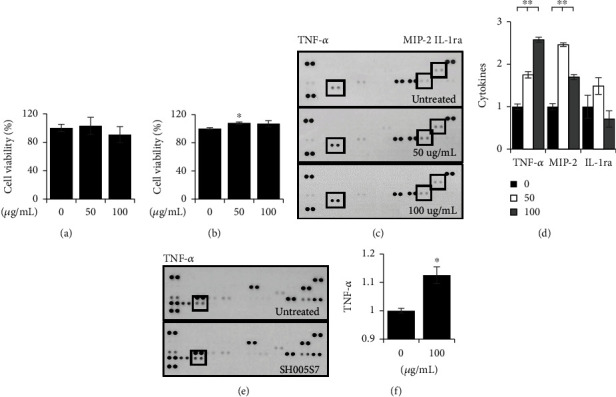
SH005S7 induces macrophage cytokines in RAW264.7 and BMDM cells. (a) RAW264.7 cells were treated with indicated concentrations of SH005S7 for 24 hours. The cell viability was then measured using MTT assay. The mean ± S.D. values are presented (*n* = 3 per group). (b) BMDM cells cultured in medium containing concentrations of SH005S7 (0, 50, and 100 *μ*g/mL) for 48 hours. Cell proliferation was quantified as the mean ± S.D. values (*n* = 3 per group; Student's *t*-test; ^∗^*p* < 0.05). (c, d) Secretion profiles of Raw264.7 macrophages in the presence of SH005S7 or not (control), as detected by a Proteome Profiler mouse cytokine array. The intensity of each spot was analyzed by ImageJ software. Statistical significance was measured by the Student's *t*-test (^∗∗^*p* < 0.01 versus control). (e, f) Cytokine profiles of the BMDM cells were obtained by performing the R&D mouse cytokine array kit. The rectangle indicates TNF-*α* cytokines showing altered expression levels in the SH005S7-treated BMDM cells, and significance was evaluated through student's *t*-test. (^∗^*p* < 0.05 versus control).

SH005S7 was formulated as herbal mixtures to provide anticancer effects and to include immune-modulatory effects. To explore the immunostimulatory effects of SH005S7, we examined the treatment of SH005S7 on the mouse macrophage cell line, RAW264.7 cells, and bone marrow-derived macrophage (BMDM). The high SH005S7 concentrations (up to 100 *μ*g/mL) used in all experiments did not induce cytotoxicity (Figures 4(a) and 4(b)). In addition, when the mouse spleen was isolated and treated with SH005S7, the proliferation effect was shown (Supplementary Figure 2). Mouse cytokine array was tested to investigate which cytokines were secreted by SH005S7 treatment. In RAW264.7 cells, SH005S7 induced macrophages to express the immune and inflammatory responses tumor necrosis factor alpha (TNF-*α*), macrophage inflammatory protein (MIP-2), and intereukin-1ra (IL-1ra) (Figures 4(c) and 4(d)). In BMDM, the expression of TNF-*α* was increased at 100 *μ*g/mL similar to the mouse macrophage cell line (Figures 4(e) and 4(f)). These results indicate that SH005S7 contributes to anticancer effects on lung cancer cells by enhancing the immune-stimulatory effects of macrophages.

#### 3.1.5. SH005S7 Has Antitumor Activity in the Animal Model

**Figure 5:**
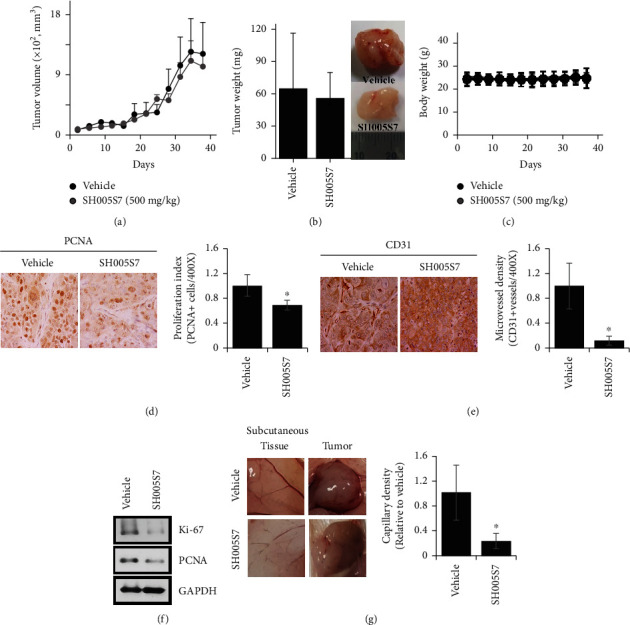
SH005S7 blocks the growth of xenographic implanted lung cancer in nude mice. HCC827GR cells (2 × 10^6^) were transplanted subcutaneously to BALB/c (nu/nu) mice. Animals were randomly assigned to each group; saline (vehicle) or SH005S7 was administered orally for 39 days. Mice were sacrificed 1 day after last administration. (a) Growth curves of tumor volume in xenograft mice. (b) Tumor weight removed from the mice. (c) Body weight during the SH005S7 oral administration period. (d, e) Representative images in tumor sections and quantitative analysis of cell proliferation marker PCNA and angiogenesis marker CD31. IHC magnification ×400. (f) Western blot analysis of proliferation marker (Ki-67 and PCNA) in HCC827GR-derived xenografts treated with vehicle or SH005S7 three times a week. (g) Macroscopic images of vascular irrigation in tumors derived from HCC827GR cells and quantitative presentation of capillary density.

**Figure 6:**
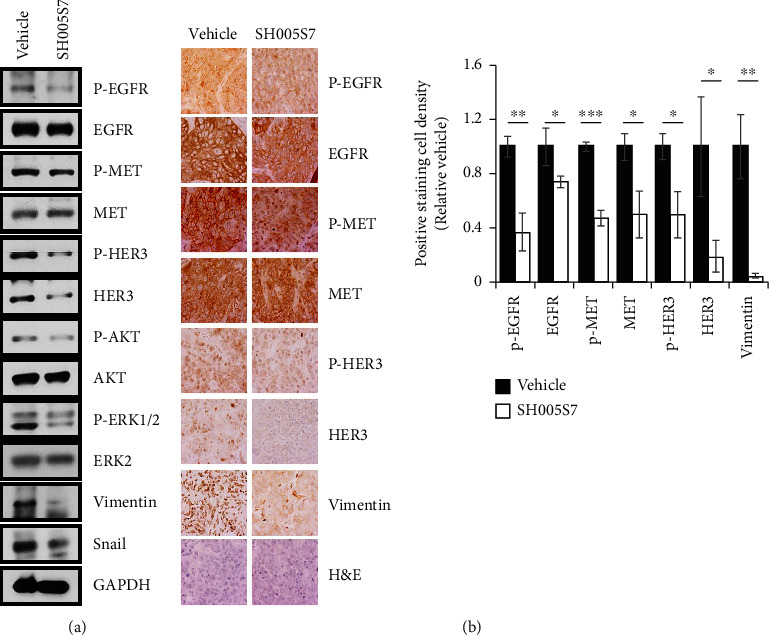
SH005S7 inhibits RTK signaling in the xenograft mouse model. (a) Western blot analysis of EMT and RTK signaling pathway in xenografts. (b) H&E and immunohistochemical staining results of RTKs on tumor sections (magnification ×400). Representative graph showing IHC quantification. The results were shown as means ± S.D. from three independent experiments. Error bars are S.D. (^∗^*p* < 0.05, ^∗∗^*p* < 0.01, and ^∗∗∗^*p* < 0.001 versus control).

The growth inhibitory effects of SH005S7 was confirmed *in vivo* using HCC827GR cell xenograft mouse models. Treatment of mice with SH005S7 for 39 days decreased growth of xenograft tumors as compared to vehicle (Figure 5(a)). There was a slight decrease in SH005S7-treated mice at the end point measurement during the oral administration period (Figure 5(b)). However, no change in body weight was observed (Figure 5(c)). To evaluate *in vivo* serum-related biomarkers for liver and kidney toxicity, the analysis was performed but there was no significant difference between groups (Table 2). Although, in CBC analysis, lymphocytes were decreased in the SH005S7-treated group, all data were within the normal range (Table 3). Next, we examined the proliferation (PCNA) and angiogenesis (CD31) markers in HCC827GR tumor tissues by IHC analysis and Western blotting. Consistent with the *in vitro* study, the results showed that SH005S7-treated tumors exhibited a significant decrease in the proliferative index (PCNA status) as well as inhibition of CD31, which is involved in new vessel formation (Figures 5(d)–5(f)). At tissue harvest, blood vessels from the surrounding tissue were observed growing into and along the implanted HCC827GR cells. As shown in Figure 5(g), the thick vessel structure was found in the vehicle, while treatment with SH005S7 notably suppressed vessel formation. We also investigated the mechanism of SH005S7 action in xenografts and observed a decrease in proliferative marker and RTK signaling pathway at the protein level (Figure 6(a)). In the IHC analysis, positive staining cells were decreased in the group treated with SH005S7 compared to the vehicle (Figure 6(b)). These findings are in agreement with *in vitro* experiments and suggest that the antitumor activity of SH005S7 on subcutaneous HCC827GR tumors is inhibition of growth.

## 4. Discussion

The aim of this study was at determining whether SH005S7 has anticancer potential by overcoming primary and acquired resistance of lung cancer cells. When examined cell lines and animal mouse model, SH0005S7 significantly inhibited the proliferation of both primary- and acquired resistance-cells (HCC827 and HCC827GR). These results indicate the antiproliferative effects of this remedy against lung cancer and gefitinib-resistant cells. We also found that SH005S7 effectively suppressed the growth of HCC827GR in a xenotropic mouse model. These results confirmed that the proliferation markers (Ki-67, and PCNA) were down-regulated by SH005S7. To our knowledge, our study is the first report to examine the anti-cancer effects on SH005S7 against both primary and acquired resistance lung cancer by using in vitro and in vivo model.

Next, when examined for the mechanism by which SH005S7 proves its effects against lung cancer in cell culture or in animal models, we found that SH005S7 is a potent inhibitor of the RTK-MEK/EGFR/HER3 activation and related gene products, which has been closely linked to proliferation and chemoresistance in NSCLC. Intracellular signaling networks comprising multiple RTKs and their downstream proteins may confer the molecular complexity and compensatory pathway against TKI-mediated inhibition of tumor cell growth [55]. In particular, members of the EGFR family, including EGFR, HER2, HER3, HER4, and MET, have been reported to communicate each other, thereby developing resistance to EGFR-TKIs [56]. Moreover, many studies suggest that MET and EGFR family-related signaling pathways play a pivotal role in the growth, invasion, angiogenesis, and metastasis of NSCLC [32–35]. First, MET is constitutively dysregulated in human cancer cells and is associated with tumor progression-related diverse signaling processes, mainly the MAPK/ERK and PI3K/AKT pathways [27–30]. In addition, acquired resistance to EGFR TKIs in NSCLC has been reported to emerge by recruiting MET and subsequent activation of HER3-AKT [9]. Second, EGFR promotes NSCLC growth through regulation of cell cycle [4–7]. Third, MET-and EGFR-regulated gene products- vimentin, snail, and MMP-9- are associated with migration, invasion, and metastasis of NSCLC cells [57]. Finally, the MET and EGFR family including HER3 enhances the angiogenic potential of NSCLC cells via increased expression of proangiogenic factors, including VEGF and CD31 [58]. Based on the crucial roles of the MET and EGFR family in MET-amplified and gefitinib-resistant NSCLC, we sought to determine whether treatment with SH005S7 could diminish these oncoproteins. We found that SH005S7 abrogated all 4 evidence-related molecule expression for the growth, invasion, angiogenesis, and metastasis of primary and acquired resistance cells against both lung cancer cell culture and animal model as indicated by immunohistochemical and Western blot analysis. Considering that SH005S7, which would be a potent RTK inhibitor, can modulate multiple targets, it merits further a multigenic disease-cancer exploration and is a rational strategy to overcome acquired chemotherapy resistance.

One of the limitations with the current chemotherapy is that most anticancer drugs suffer from hepatotoxicity and nephrotoxicity. When administered orally to nude mice xenografically transplanted human NSCLC, SH005S7 did not exhibit hepatotoxicity and nephrotoxicity in animals and had no change in body weight except inhibiting the growth of the tumors. Therefore, these data demonstrate the substantial level of SH005S7 in lung cancer after oral administration and confirm the bioavailability of SH005S7 and correlate with the suppression of tumor volume.

During the evolution of neoplastic diseases, immune macrophage functions are usually attenuated and this presents a problem to therapies against cancer. Macrophages are phagocytic cells of the innate immune system that are located in various tissues and enhance the immune response by elimination of apoptotic cells and pathogens secreting cytokines such as TNF-*α* [59]. Here, we investigated the effects of SH005S7 on immune stimulatory response in macrophage cells. TNF-*α* cytokine release of SH005S7 on macrophages, RAW 264.7 cells, and BMDM was significantly increased, without cell cytotoxicity. Additionally, when doing treatment of SH005S7, the proliferation effect was shown in splenocyte, the main filter for blood-borne pathogens and antigens, and a secondary lymphoid organ for immune and hematopoietic functions [60]. This study suggests that SH005S7 enhances the immune response by stimulating the macrophage and the spleen.

However, there are limitations of the current study on how SH005S7 can inhibit the kinase activity and constitutive overexpression of wild and mutant EGFR or Met that may target mutant receptor conformations by selective, degradation of these and so on thereby modulating the EGFR or Met downstream signaling pathways. Therefore, further experiments are needed.

## 5. Conclusions

Taken together, our results overall suggest that SH005S7 can inhibit the growth of human primary and chemoresistance lung cancer in cells and in animal mice by inhibiting various biomarkers of the disease, leading to the inhibition of proliferation. Of note, there is a possibility that SH005S7, a new formula herbal remedy, may substitute for EGFR family related multiple-targeted chemotherapeutic agents as a part of lung cancer treatment.

## Figures and Tables

**Table 1:** Contents of standard compounds in SH005S7.

Scientific names	Latin binomial nomenclature	Standard compound	SH005S7 at 500 *μ*g/mL
*Rhus verniciflua* Stokes	Lacca Sinica Exsiccata	Fisetin	6.37 *μ*g (1.274%)
*Angelica gigas* Nakai	Angelicae Gigantis Radix	Decursin	0.27 *μ*g (0.054%)
*Morus alba* L.	Mori Radicis Cortex	Morusin	0.093 *μ*g (0.019%)

**Table 2:** Results of *in vivo* serum analysis.

	Control	SH005S7
ALP	155.0 ± 53.1	96.7 ± 45.5
ALT	22.2 ± 12.1	32.3 ± 29.9
Creatinine	0.8 ± 0.4	0.5 ± 0.2

Data are means ± S.D. ALP: alkaline phosphatase; ALT: alanine aminotransferase.

**Table 3:** Results of *in vivo* CBC analysis.

Parameter	Control	SH005S7
WBC (K/*μ*L)	5.44 ± 4.75	5.13 ± 4.70
NE (K/*μ*L)	3.50 ± 4.51	1.86 ± 1.64
LY (K/*μ*L)	1.48 ± 0.43	0.88 ± 0.29^∗^
MO (K/*μ*L)	0.30 ± 0.21	0.19 ± 0.10
EO (K/*μ*L)	0.12 ± 0.11	0.08 ± 0.06
BA (K/*μ*L)	0.03 ± 0.03	0.02 ± 0.06
RBC (M/*μ*L)	8.93 ± 1.91	7.22 ± 1.88
Hb (g/dL)	12.46 ± 2.94	10.27 ± 2.95
HCT (%)	39.82 ± 9.38	32.43 ± 8.31
MCV (fL)	44.44 ± 1.28	45.10 ± 1.95
MCH (pg)	13.88 ± 0.58	14.13 ± 0.77
MCHC (g/dL)	31.26 ± 1.31	31.35 ± 1.58
RDW (%)	18.36 ± 1.42	17.17 ± 1.20
PLT (K/*μ*L)	532.40 ± 72.20	317.00 ± 242.85
MPV (fL)	3.62 ± 0.25	3.58 ± 0.44

Data are means ± S.D. ^∗^Significantly different compared to control, ^∗^*p* < 0.05. BA: basophils; CBC: complete blood count; EO: eosinophils; Hb: hemoglobin; HCT: hematocrit; LY: lymphocytes; MCH: mean corpuscular hemoglobin; MCHC: mean corpuscular hemoglobin concentration; MCV: mean corpuscular volume; MO: monocytes; MPV: mean platelet volume; NE: neutrophils; PLT: platelet; RBC: red blood cell; RDW: red cell distribution; WBC: white blood cell.

## Data Availability

The data used to support the findings of this study are included within the article.

## Supplementary Materials

Supplementary Materials Supplementary data associated with this article can be found in the Supporting Information. Supplementary Figure 1: HCC827GR cell resistance to gefitinib treatment. (a) HCC827 and HCC827GR cells were treated with gefitinib (0.5, 1, 2, and 5 *μ*M) in 96-well plates for 24 hours, and the cell viability was examined by MTT assay. (b) RTK signaling pathways were detected in HCC827 and HCC827GR cells by Western blotting assay. All results are from three independent experiments. Supplementary Figure 2: SH005S7 induces cell proliferation in mouse splenocytes. Proliferation effects of SH005S7 on mice splenic lymphocytes at different concentrations (0, 50, 100, 200, and 500 *μ*g/mL) for 48 hours determined via MTT assay. The results were shown as means ± S.D. from three independent experiments.
