# Supplementary figures and images for: SH005S7 Overcomes Primary and Acquired Resistance of Non-Small Cell Lung Cancer by Combined MET/EGFR/HER3 Inhibition
Source: Biomed Res Int. 2022 Sep 15;2022:1840541. doi: 10.1155/2022/1840541 (PMC9499774; doi:10.1155/2022/1840541)

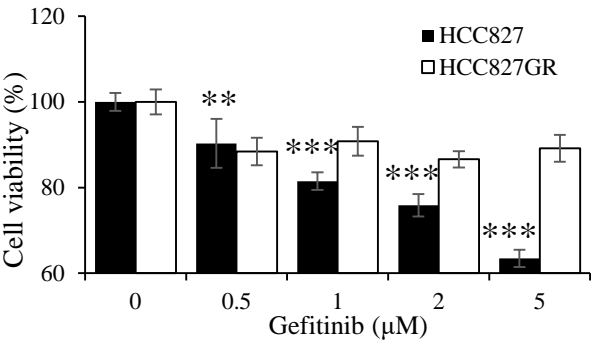

(a)

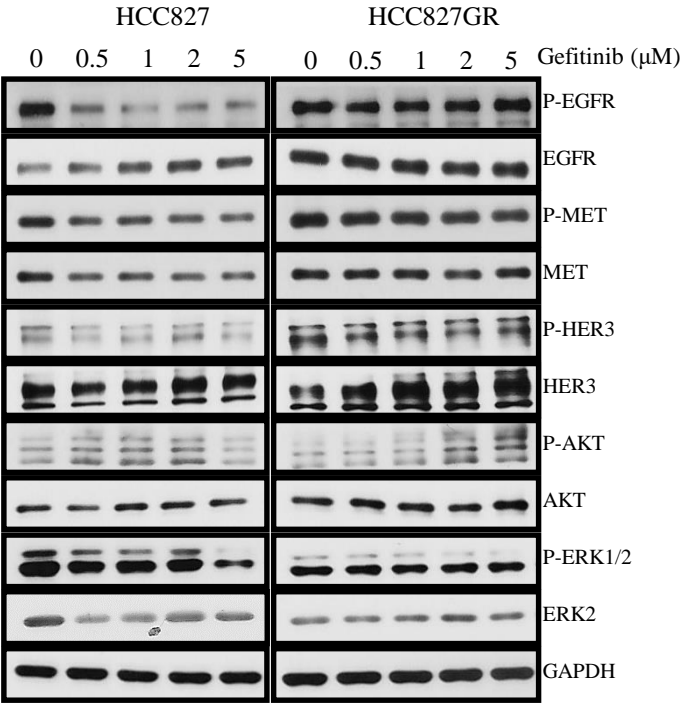

(b)

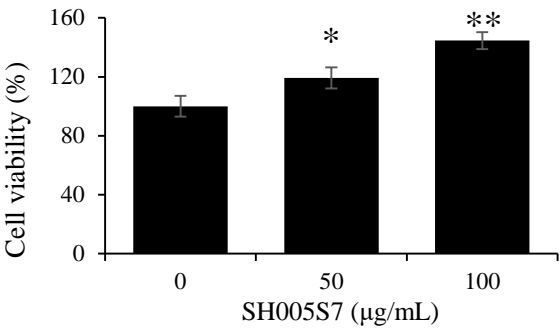

Supplement: Supplementary Materials — Supplementary data associated with this article can be found in the Supporting Information. Supplementary Figure 1: HCC827GR cell resistance to gefitinib treatment. (a) HCC827 and HCC827GR cells were treated with gefitinib (0.5, 1, 2, and 5 μM) in 96-well plates for 24 hours, and the cell viability was examined by MTT assay. (b) RTK signaling pathways were detected in HCC827 and HCC827GR cells by Western blotting assay. All results are from three independent experiments. Supplementary Figure 2: SH005S7 induces cell proliferation in mouse splenocytes. Proliferation effects of SH005S7 on mice splenic lymphocytes at different concentrations (0, 50, 100, 200, and 500 μg/mL) for 48 hours determined via MTT assay. The results were shown as means ± S.D. from three independent experiments. [file 1840541.f1.pdf]
